# Microbiota Dynamics in Patients Treated with Fecal Microbiota Transplantation for Recurrent *Clostridium difficile* Infection

**DOI:** 10.1371/journal.pone.0081330

**Published:** 2013-11-26

**Authors:** Yang Song, Shashank Garg, Mohit Girotra, Cynthia Maddox, Erik C. von Rosenvinge, Anand Dutta, Sudhir Dutta, W. Florian Fricke

**Affiliations:** 1 Institute for Genome Sciences, University of Maryland School of Medicine, Baltimore, Maryland, United States of America; 2 Division of Gastroenterology, Sinai Hospital of Baltimore, Baltimore, Maryland, United States of America; 3 Division of Gastroenterology and Hepatology, University of Maryland School of Medicine, Baltimore, Maryland, United States of America; 4 Department of Medicine, University of Maryland School of Medicine, Baltimore, Maryland, United States of America; Graz University of Technology (TU Graz), Austria

## Abstract

*Clostridium difficile* causes antibiotic-associated diarrhea and pseudomembraneous colitis and is responsible for a large and increasing fraction of hospital-acquired infections. Fecal microbiota transplantation (FMT) is an alternate treatment option for recurrent *C. difficile* infection (RCDI) refractory to antibiotic therapy. It has recently been discussed favorably in the clinical and scientific communities and is receiving increasing public attention. However, short- and long-term health consequences of FMT remain a concern, as the effects of the transplanted microbiota on the patient remain unknown. To shed light on microbial events associated with RCDI and treatment by FMT, we performed fecal microbiota analysis by 16S rRNA gene amplicon pyrosequencing of 14 pairs of healthy donors and RCDI patients treated successfully by FMT. Post-FMT patient and healthy donor samples collected up to one year after FMT were studied longitudinally, including one post-FMT patient with antibiotic-associated relapse three months after FMT. This analysis allowed us not only to confirm prior reports that RCDI is associated with reduced diversity and compositional changes in the fecal microbiota, but also to characterize previously undocumented post-FMT microbiota dynamics. Members of the *Streptococcaceae*, *Enterococcaceae*, or *Enterobacteriaceae* were significantly increased and putative butyrate producers, such as *Lachnospiraceae* and *Ruminococcaceae* were significantly reduced in samples from RCDI patients before FMT as compared to post-FMT patient and healthy donor samples. RCDI patient samples showed more case-specific variations than post-FMT patient and healthy donor samples. However, none of the bacterial groups were invariably associated with RCDI or successful treatment by FMT. Overall microbiota compositions in post-FMT patients, specifically abundances of the above-mentioned Firmicutes, continued to change for at least 16 weeks after FMT, suggesting that full microbiota recovery from RCDI may take much longer than expected based on the disappearance of diarrheal symptoms immediately after FMT.

## Introduction

*Clostridium difficile*, the pathogen associated with the majority of infective antibiotic-associated diarrhea and causative agent of pseudomembraneous colitis [1], is responsible for a large fraction of nosocomial, or hospital-acquired, disease [2]. Today, in parts of the U.S., the incidence of infections with *C. difficile* is higher than that of methicillin-resistant *Staphylococcus aureus* [3]. *C. difficile* infection (CDI) is believed to result from gastrointestinal dysbiosis, i.e., the disruption of the resident microbiota, often caused by antibiotic treatment, which enables *C. difficile* to establish an infection. *C. difficile* can be acquired via fecal-oral transmission of spores that survive atmospheric oxygen and gastric acid exposure and germinate in the large intestine. However, carriage of *C. difficile* is not always associated with disease, as asymptomatic *C. difficile* colonization is well recognized [4], especially in newborns and infants of <1 year age [5].

Besides treatment with almost any antibiotic [6]–[14], other factors associated with increased risk for *C. difficile* infection include old age, recent hospitalization, tube feeding, use of gastric acid-suppressing drugs and underlying chronic disease, including inflammatory bowel disease [15]–[19]. Recent evidence suggests that excessive inflammatory responses in the human host enhance the severity of CDI [20].

Standard treatment for *C. difficile* infection consists of metronidazole or vancomycin administration and, more recently, fidaxomicin. However, the rate of recurrent *C. difficile* infection (RCDI) after initial therapy is about 20% [21] and even higher after subsequent antibiotic courses and recurrences [8], [22]. Consequently, despite current therapeutic options, RCDI treatment has become increasingly challenging and the incidence of RCDI has been rising during the past decade resulting in increased healthcare cost and significant morbidity [23].

Fecal microbiota transplantation (FMT), which aims to restore a normal, functional intestinal microbiota from a healthy donor in the RCDI patient, has recently received increasing attention in clinical and research communities [24]–[27] and has also become a popular subject of discussion in other media. First documented in the fourth century in China and in 1958 in the U.S., FMT was shown in a recent systematic review of 317 patients in 27 separate studies to have an overall success rate of 92% [28]. The exact mechanism of action responsible for the success of FMT to treat RCDI remains unknown and there is no clinically validated set of parameters to define a suitable donor or ideal donor microbiota, although attempts in this direction have been made [29]. Short- and long-term effects of FMT on the recipient microbiota remain a concern, especially in light of the growing body of literature that implicates the gastrointestinal microbiota in a large number of diseases [30]. For the same reason, there is significant clinical interest in therapeutic options to target the microbiota to treat microbiota-associated health problems besides RCDI. As a result, attempts to treat IBD [31]–[33], metabolic syndrome [34] and other diseases [35], [36] by FMT have been made.

Clinical concerns and the increasing number of FMT procedures performed by U.S. physicians recently led the U.S. Food and Drug Administration (FDA) to release new guidelines that define FMT as a biologic therapy that requires physicians to obtain an investigational new drug (IND) application [37]. Shortly after this guideline was a released, however, the FDA announced a decision to exercise enforcement discretion in order to allow physicians to perform FMT in patients with RCDI not responsive to standard therapy. The urgency for further research into the short- and long-term effects of FMT is highlighted by the fact that the public awareness of FMT as a treatment option for RCDI has increased to a degree where do-it-yourself protocols have become available over the Internet and the procedure is being performed without medical surveillance.

In this study, we applied 16S rRNA amplicon pyrosequencing to analyze fecal samples from RCDI patients and their corresponding donors before and after FMT. For the first time, we included longitudinal simultaneous sampling of both post-FMT patients and healthy donors for up to one year after FMT. This unique sample set allowed us to describe previously undocumented microbiota dynamics in post-FMT patients after resolution of CDI. In addition, inclusion of a patient, who was initially treated successfully by FMT but experienced relapse after new antibiotic treatment, provided us with the unique opportunity to distinguish microbiota changes seen in a previously asymptomatic patients after relapse of CDI from those apparent in RCDI patients with long-term disease and multiple courses of anti-*C. difficile* antibiotic treatment.

## Materials and Methods

### Study cohort and sample collection

The Institutional Review Board of Sinai Hospital Baltimore approved the study under protocol number #1826 and all subjects provided their written informed consent to participate in the study. FMT was performed at Sinai Hospital of Baltimore, Baltimore, MD by infusion of a fecal solution prepared by a predefined protocol (Dutta et al., submitted) based on Aas et al. [38]. Potential donors were thoroughly clinically evaluated based on history, physical examination and serological screening for HIV, syphilis, hepatitis A, B and C and *Helicobacter pylori* infection. Fecal specimens of patients and donors were tested 3–5 days before FMT for the presence of pathogenic bacteria (salmonella, shigella, yersinia), parasites (entamoeba, giardia, worms), and *C. difficile*. Patients were admitted to the hospital the day before and bowel prep administered the night before FMT. Patients were also administered a proton pump inhibitor (omeprazole, 20 mg) on the evening and morning before the procedure. Donor fecal samples (25–30 g) were mixed with 250 ml of sterile saline buffer, mixed into slurry and filtered once with surgical gauze for large particles and twice with a coffee filter. The volume of the filtrate was increased to 450 ml with sterile saline buffer and divided into 5 aliquots of 90 ml. For FMT, two aliquots (180 ml) were endoscopically delivered by spray catheter into the jejunum. The remaining three aliquots were instilled by colonoscopy into the right colon (180 ml) and transverse and upper descending colon (90 ml).

**Figure 1:**
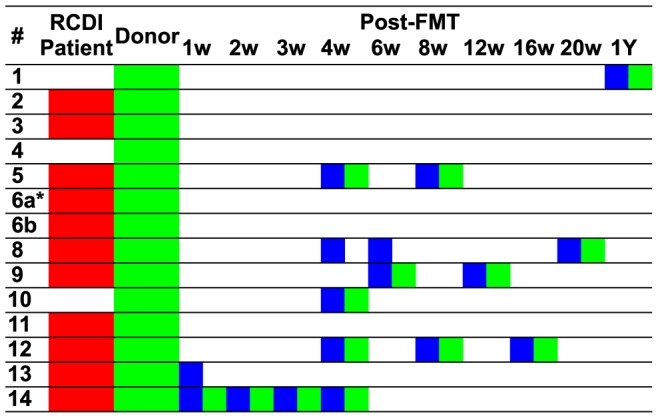
Overview of analyzed patient and donor samples. RCDI patient samples are marked in red, post-FMT patient samples in blue and donor samples in green. *Patient #6a experienced antibiotic-induced relapse of *C. difficile* infection and was treated successfully with a second round of FMT as patient #6b. In the NCBI short read archive, samples referred to as #6b are designated as #7 samples.

The clinical aspects of this study, including a comprehensive description and discussion of the FMT-treated patient population and individual case metadata, are provided in a separate publication (Dutta et al., submitted). Fecal samples were collected from 14 patient-donor pairs and used for this study (Fig. 1; Table 1). All patients had at least three recurrences of *C. difficile* infection and were treated with at least three courses of antibiotics. Fecal samples were collected before and after FMT from patients and, at corresponding time points, from their respective donors, which included family members (spouses and children) and friends (Fig. 1).

**Table 1:** RCDI patient study population.

Case [#]	Sex	Age	RCDI duration [months]	Donor	Time to resolution of symptoms [days]	Follow up [months]	Inciting antibiotic
1	F	65	18	Husband	2	26	Beta-lactam^1^ + lincosamide^2^
2	F	65	6	Husband	3	21	multiple
3	F	61	5	Friend	2	22	Lincosamide^2^
4	F	56	12	Friend	3	19	Fluoroquinolones
5	F	76	72	Friend	2	7	Fluoroquinolones
6a*	F	57	8	Son	3	18	Fluoroquinolones
6b*			2	Brother	4		Fluoroquinolones^3^
8	F	72	5	Daughter	3	17	Unknown
9	F	63	6	Husband	2	17	Lincosamide^2^ + fluoroquinolone^4^
10	F	61	11	Husband	3	17	Clindamycin
11	M	68	6	Wife	3	16	Unknown
12	F	41	12	Husband	2	16	Lincosamide^2^
13	F	79	12	Husband	3	12	Unknown
14	M	57	4.5	Wife	2	12	Unknown

* #6a had a relapse of RCDI one month after successful FMT and received a second FMT three months after the first (#6b). In the NCBI short read archive, samples referred to as #6b are designated as #7 samples.

1 Penicillin; ^2^ clindamycin; ^3^ ciprofloxacin; ^4^ levofloxacin.

### Sample collection and nucleic acid isolation

All fecal samples were self-collected by patients and donors without bowel preps, stored in the freezer and within 24 hours brought to Sinai Hospital, after which they were stored at –80°C. Patients stopped antibiotic use 5 days before the FMT procedure; RCDI patient samples were taken 1–2 days prior to FMT. For processing, samples were thawed at 4°C and in aliquots of 0.15 g per tube re-suspended in 1 ml of 1 × phosphate-buffered saline. Cell lysis was initiated with two enzymatic incubations, first using 5 µl of lysozyme (10 mg ml^−1^; Amresco, Solon, OH, USA), 13 µl of mutanolysin (11.7 U µl^−1^; Sigma-Aldrich) and 3 µl of lysostaphin (4.5 U µl^−1^; Sigma-Aldrich) for an incubation of 30 min at 37°C and, second, using 10 µl Proteinase K (20 mg ml^−1^; Research Products International, Mt Prospect, IL, USA), 50 µl 10% SDS and 2 µl RNase (10 mg ml^−1^) for an incubation of 45 min at 56°C. After the enzyme treatments, cells were disrupted by bead beating in tubes with Lysing Matrix B (0.1 mm silica spheres, MP Biomedicals, Solon, OH, USA), at 6 m s^−1^ for 40 s at room temperature in a FastPrep-24 (MP Biomedicals). The resulting crude lysate was processed using the ZR Fecal DNA mini-prep kit (Zymo, Irvine, CA, USA) according to the manufacturer’s recommendation. The samples were eluted with 100 µl of ultra pure water into separate tubes. DNA concentrations in the samples were measured using the Quant-iT PicoGreen dsDNA assay kit (Molecular Probes, Invitrogen, Carlsbad, CA, USA).

### Amplification and sequencing

In brief, hypervariable regions V1–V3 of the bacterial 16S rRNA gene were amplified with primers 27F and 534R as described previously [39]. DNA amplification of 16S rRNA genes was performed using AccuPrime *Taq* DNA polymerase High Fidelity (Invitrogen) and 50 ng of template DNA in a total reaction volume of 25 µl, following the AccuPrime product protocol. Reactions were run in a PTC-100 thermal controller (MJ Research, Waltham, MA, USA) using the following protocol: 3 min of denaturation at 94°C, followed by 30 cycles of 30 s at 94°C (denaturation), 30 s at 52°C (annealing) and 45 ss at 68°C (elongation), with a final extension at 68°C for 5 min.

Equimolar amounts (50 ng) of the PCR amplicons were mixed in a single tube. Amplification primers and reaction buffer were removed using the AMPure Kit (Beckman Coulter, Brea, CA, USA) and purified amplicon mixtures sequenced at the Institute for Genome Sciences, University of Maryland, using 454 primer A and protocols recommended by the manufacturer (Roche, Branford, CT, USA). Raw sequences were deposited in the Short Read Archive Database (http://www.ncbi.nlm.nih.gov/sra; project number SRP016902). In the NCBI short read archive, samples referred to as #6a are designated as #6 samples and samples referred to as #6b as #7 samples.

### Sequence processing and analysis

16S rRNA sequence reads were processed with QIIME [40] and CloVR [41], using the automated CloVR-16S pipeline as described in the corresponding standard operating procedure [42]. Briefly, using the QIIME split_libraries.py tool sequences were binned based on sample-specific barcodes, trimmed by removal of barcode and primer sequences and filtered for quality, using the default parameters, except for "—barcode-type "variable_length". Chimeric sequences were removed with UCHIME [43] using MicrobiomeUtilities (http://microbiomeutil.sourceforge.net/) and the rRNA16S.gold.fasta reference database. Reads were clustered into operational taxonomic units (OTUs) using a similarity threshold of 95%. On average, OTUs were classified using the RDP Naive Bayesian Classifier [44] with a score filtering threshold of 0.5. Rarefaction curves were calculated based on OTU counts using the rarefaction.single routine of the Mothur package [45]. Hierarchical clustering, boxplots, and statistical calculations (Wilcoxon rank sum tests, Jensen-Shannon divergence etc.) were performed in R. Differentially abundant OTUs were determined with Metastats [46]. Phylogenetic trees were created with FastTree2 [47] using trimmed alignments generated with NAST. Dot plots to evaluate phylogenetic distances and Jensen-Shannon divergence between sample pairs and changes in relative abundance of specific taxonomic families over time were generated with Prism5 (version 6 for Mac, GraphPad Software, San Diego CA, USA).

## Results and Discussion

### Patient population, sample set and sequence data

For this longitudinal study, fecal samples were collected from 14 pairs of RCDI patients, treated successfully by FMT, and their respective donors (Fig. 1). In addition to the 14 donor samples used for FMT, 11 samples from pre-FMT RCDI patients and 17 samples from eight post-FMT patient samples, as well as 14 samples from eight healthy donors collected after FMT were analyzed, collected between one week and one year after the procedure, (total number of samples: 56). This allowed us to perform the first characterization of long-term microbiota changes in patients after FMT. All treated RCDI patients experienced resolution of diarrheal symptoms within 2–3 days after FMT (Table 1), in accordance with previous reports [27]. Of the post-FMT samples collected from asymptomatic patients, 14 were paired with donor samples collected at the same time points to serve as a control for intra-individual, longitudinal variations not associated with RCDI. RCDI patient #6a was successfully treated by FMT but experienced recurrence of *C. difficile* infection one month later, after being treated for a urinary tract infection with ciprofloxacin. Subsequent oral vancomycin and intravenous immunoglobulin therapy did not resolve the problem. The patient #6a was treated successfully for a second time by FMT, three months after the first FMT (designated as case #6b). Selected characteristics of all cases for which samples were analyzed are summarized in Table 1. Additional clinical aspects of this study have been described in a separate publication [48] FMT donors for this study were chosen by the RCDI patients and included genetically unrelated individuals living in the same household (8x spouses), as well as genetically related (2x children) or unrelated (3x friends) individuals living in households separate from those of the RCDI patients (Table 1). On average, 3,315 sequence reads were obtained per sample using the Roche/454 GS FLX Titanium platform (average sequence length: 527 bp). A list of read numbers and identified operational taxonomic units (OTUs) for each of the samples is part of the supplement (Table S1).

### Reduced microbiota diversity in RCDI patients increases after FMT

**Figure 2:**
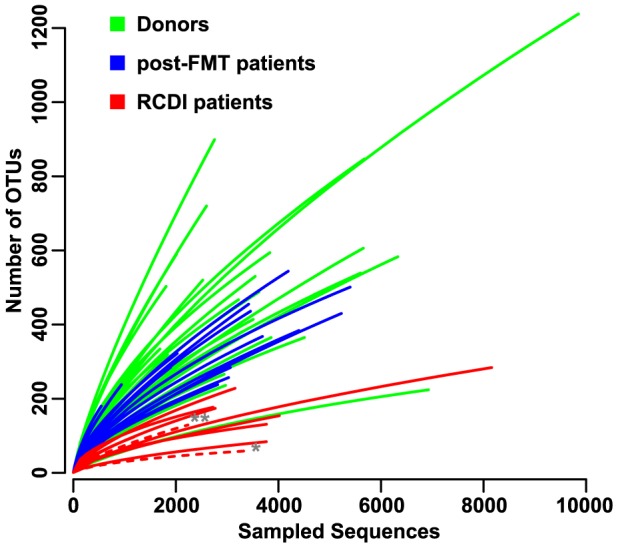
Microbiota rarefaction curves showing fecal microbiota diversity in RCDI (red) and post-FMT (blue) patient and donor (green) samples. Each curve shows the average number of OTUs found in a given number of sampled sequences. OTUs can be treated as equivalent to taxonomic species in the sequence space. RCDI samples are marked from patient #6a (*), who experienced antibiotic-induced relapse and was treated by FMT again as patient #6b (**).

**Figure 3:**
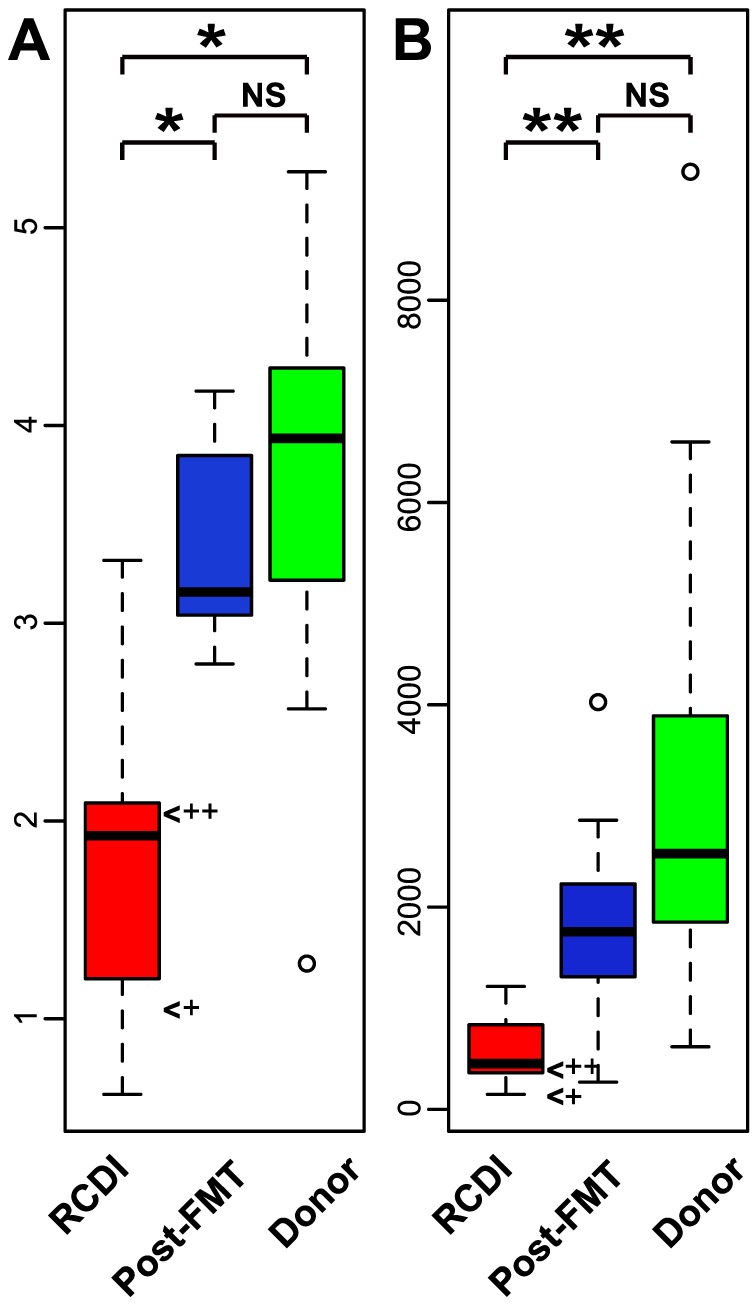
Microbiota diversity (Shannon) and richness (ACE) of RCDI and post-FMT patient and donor samples. (A) Shannon index; (B) ACE index. Significant differences are shown (*, p<0.01; **, p<0.001) as measured by Wilcoxon rank sum test. RCDI samples from patient #6a (+), who experienced antibiotic-induced relapse and was treated by FMT again as patient #6b (++) are marked.

Reduced microbiota diversity associated with *C. difficile* infection is reported in humans [49]-[51] and mice [52], [53]. This finding was confirmed in our study with multiple post-FMT samples collected up to one year after the procedure. Compared to healthy donors the fecal microbiota diversity of RCDI patients was reduced, as shown by rarefaction analysis of OTU counts (Fig. 2). Microbiota diversity increased significantly in post-FMT patient samples, as demonstrated by Shannon diversity index calculations (p<0.01, Wilcoxon rank sum test) between RCDI (mean 1.68± 0.75) and post-FMT (mean 3.37± 0.46) patient samples (Fig. 3). Microbial richness was also increased in post-FMT compared to RCDI patient samples, based on the comparison of mean ACE indices (46%; p < 0.001). Interestingly, no significant difference in microbial diversity or richness was noted between post-FMT patient and donor samples as determined by Shannon and ACE indices. Shannon diversity increased in all 17 post-FMT patients as soon as one week after FMT and remained stable and comparable among different patients for up to one year afterwards (Fig. S1).

Compared to the RCDI sample collected before the first FMT treatment (#6a_P0), microbial diversity in the RCDI sample from the same patient collected three months later after RCDI relapse (#6b_P0) showed a 2-fold increase based on the Shannon index but was still low compared to healthy donor samples (Fig. 3). These results suggest that FMT restores the reduced microbiota diversity associated with RCDI. Furthermore, diversity increases immediately after FMT and remains stable over time.

### FMT shifts fecal microbiota towards healthy donor composition

To gain further insights into the effects of FMT on the patient microbiota, shared OTUs between RCDI patients, post-FMT patients and healthy donor samples were determined (Fig. S2). Using a threshold of at least five supporting reads across all 38 samples for OTUs to be considered in the comparison, a total of 1,321 OTUs were identified of which 876 (65%) were only identified in post-FMT patient and healthy donor samples but never in RCDI patient samples. This finding could be interpreted to indicate that post-FMT patients acquired donor OTUs as a consequence of FMT. However, the applied analysis has a detection limit of approximately 0.03% and does not allow for the distinction of different bacterial strains from the same OTU. It is therefore impossible to distinguish between OTUs that might have been present in RCDI patients below the detection limit and those that were acquired from the donors.

**Figure 4:**
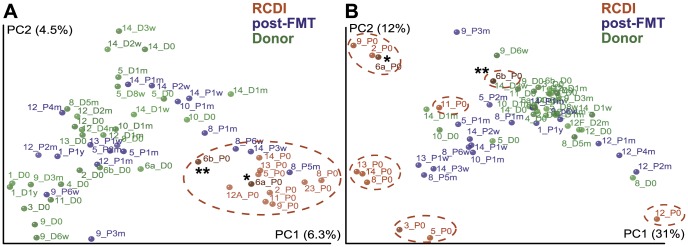
Unscaled principal coordinate analysis (PCoA) plots showing unweighted (A) and weighted (B) UniFrac analysis of RCDI (red) and post-FMT (blue) patient and healthy donor (green) samples. RCDI patient samples are circled in red. RCDI samples from patient #6a (*), who experienced antibiotic-induced relapse and was treated by FMT again as patient #6b (**) are marked in dark red. Sample names indicate case numbers, patient or donor source and time point of collection ("0" time point refers to pre-FMT sampling time points; other time points are abbreviated as weeks [w], months [m] and year [y]).

Microbiota compositions were analyzed based on phylogenetic distance calculations between samples using the unweighted, i.e., comparing OTU presences/absences, and weighted, i.e., including quantitative information about detected OTUs, UniFrac metric (Fig. 4). Principal coordinate analyses (PCoA) of the unweighted UniFrac comparison showed that most of the compositional variation among samples is accounted for by post-FMT patient and healthy donor samples (Fig. 4A). In contrast, when OTU abundance is also taken into consideration (weighted UniFrac analysis) most of the variation within the entire sample set is observed among RCDI patient samples (Fig. 4B), suggesting that relative abundances of major microbiota members can vary substantially not only between RCDI patient and healthy donor samples but also among different RCDI patient samples.

In most cases, FMT resulted in the adoption of a fecal microbiota composition in post-FMT samples that was similar to that of healthy donors. This is apparent in the clustering of post-FMT patient and healthy donor samples in unweighted UniFrac analysis (Fig. 4A). However, several patients appeared to at least temporarily return to pre-FMT fecal microbiota composition states (e.g., Patient #8 at 5 months and Patient #14 at 3 weeks after FMT), although all treated patients were reported to be symptom-free within 2–3 days after FMT. The adoption of a fecal microbiota composition in post-FMT patient samples similar to that of healthy donors was also supported by comparing mean phylogenetic UniFrac distances. These were significantly larger between RCDI and post-FMT patient samples than between post-FMT and donor samples both in unweighted (p<0.05) and weighted (p<0.01) UniFrac analysis.

Interestingly, the RCDI sample from the patient (#6a/b), who relapsed after unrelated antibiotic treatment, showed a microbiota composition that was similar to that of other post-FMT and healthy donor samples, especially in the weighted UniFrac analysis (Fig. 4). This second RCDI episode lasted only two months and included treatment with a single antibiotic (vancomycin) compared to 4.5–72 months duration and at least three different antibiotic treatments in other RCDI patients, It is therefore possible that several of the phenotypes observed in other RCDI samples are reflective of long-term disease and multiple antibiotic treatment courses. The data presented here suggest that RCDI is associated with the presence or absence of specific fecal microbiota members (i.e., co-clustering of all RCDI samples in unweighted UniFrac analysis, including #6b_P0), rather than significant changes in the relative abundance of major microbiome components (i.e., separate clustering of different RCDI samples and of #6b_P0 with healthy donor samples in weighted UniFrac analysis), which could represent a consequence of long-term disease.

### FMT affects predominantly Firmicutes and Proteobacteria

**Figure 5:**
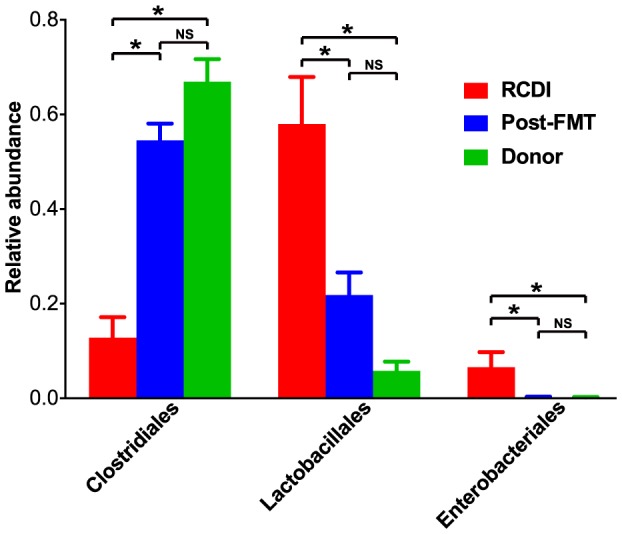
Microbiota changes between RCDI and post-FMT patient and healthy donor sample groups at the taxonomic order level. Significant differences between sample groups as calculated with the Metastats tool are marked with asterisks (p<0.01).

The identification of specific microbiota members associated with RCDI and successful FMT treatment bears the potential to identify new diagnostic markers to predict susceptibility to *C. difficile* infection or infection relapse in at-risk populations. In addition, this knowledge may provide the insights required to assemble culture-based "probiotic" bacterial mixtures as substitutes for transplantation of fecal samples, as has recently been demonstrated in humans [54] and the mouse model [55]. Towards this goal, the relative abundances of all identified microbial taxa were compared between RCDI and post-FMT patient and healthy donor sample groups using Metastats [46]. Among these three groups, bacteria from only three taxonomic orders, belonging to two phyla, showed significant changes, i.e., Clostridiales and Lactobacillales (both from phylum Firmicutes) and Enterobacteriales (phylum Proteobacteria) (Fig. 5). Clostridiales, which include the species *C. difficile,* were present at only 12.8% in RCDI patient samples and significantly increased in post-FMT samples (55%) but still remained lower compared to healthy donor samples (70%) (p<0.001, unpaired t-test with unequal variance). Lactobacillales, which were present at high abundance in RCDI patient samples (mean: 58%), were significantly decreased in post-FMT patient (22%) and healthy donor (5%) samples. However, abundance of Lactobacillales remained higher in post-FMT patient compared to donor samples (p<0.01). Enterobacteriales, present at 6.5% in RCDI patient samples, were less than 1% in post-FMT patient and donor samples (p<0.001).

**Figure 6:**
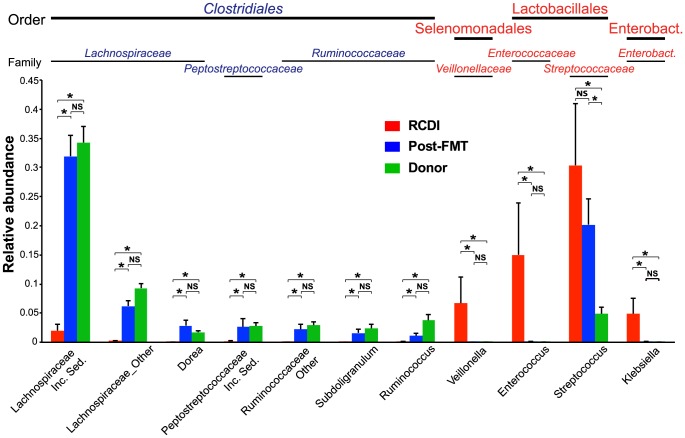
Microbiota changes between RCDI and post-FMT patient and healthy donor sample groups at the taxonomic family and genus levels. Significant differences between sample groups as calculated with the Metastats tool are marked with asterisks (p<0.01). Note that standard deviations are smaller for genera that increased in post-FMT relative to RCDI patient samples (e.g., *Lachnospiraceae* Incertae Sedis) compared to those that decreased (e.g. *Streptococcus*), which reflects differences in the relative abundances of major microbiota members among RCDI patient samples.

Three taxonomic families within the order Clostridiales (phylum: Firmicutes) significantly increased in relative abundance between RCDI and post-FMT patient samples (p<0.01), *Lachnospiraceae, Peptostreptococcaceae,* and *Ruminococcaceae* (Fig. 6). Most prominently, an uncharacterized genus within the *Lachnospiraceae* family (*Lachnospiraceae* Incertae Sedis) increased from on average 3% in RCDI patient samples to 30% in post-FMT patient samples and was 39% in healthy donor samples (p<0.01). The dominant OTU within this genus (99% identical to GenBank Acc.-No.: EF399262) was identified in all 28 donor samples (27 samples with >4 reads), 15 out of 17 post-FMT patient samples (14 samples with >4 reads), and 8 out of 11 RCDI patient samples (#6b was the only sample with >4 reads). *C. difficile* is a member of the *Peptostreptococcaceae* [56], which increased in patients after FMT. Moreover, an unknown genus within this family accounts for >2% of the fecal microbiota in healthy donor samples (Fig. 6), demonstrating that taxonomically close relatives of *C. difficile* exert non-pathogenic or even beneficial functions in the healthy intestinal microbiota.

Within the orders Lactobacillales (phylum: Firmicutes) and Enterobacteriales (phylum: Proteobacteria), the genera *Enterococcus* and *Klebsiella*, which were present on average at 18% and 4% in RCDI patient samples, respectively, were significantly reduced to less than 0.1% in post-FMT patient samples (p<0.01). Members of the *Streptococcaceae* (phylum: Firmicutes), the dominant taxonomic family in RCDI patient samples (mean: 30.1%), were reduced on average by more than 10% after FMT, although this change was not statistically significant due to large variations between RCDI patients. With the exception of the genus *Streptococcus*, none of these families or genera showed significant differences in relative abundance between post-FMT patient and healthy donor samples (p<0.05). *Streptococcus* was the only genus with a significant difference in relative abundance between both RCDI patient and donor samples and between post-FMT patient and donor samples. As post-FMT patients appear to show increased susceptibility to *C. difficile* infection compared to healthy donors, if additional antibiotic medication to treat unrelated infections becomes necessary [27], the increased abundance of the *Streptococcus* genus in this population could play a role for this susceptibility. However, not all RCDI samples contained high counts of *Streptococcus* sequences (range: 0.1% to 82.4%). In general, different RCDI samples showed more variation in the abundance of microbiota members that were increased relative to healthy donors (e.g., *Enterococcaceae* and *Streptococcaceae*) than of microbiota members that were reduced (see error bars in Fig. 6). This may suggest that the second group provides a better target for the identification of diagnostic markers for RCDI (e.g., among the *Lachnospiraceae, Peptostreptococcaceae,* and *Ruminococcaceae*).

In contrast to all other cases, the fecal RCDI microbiota from patient #6b, who experienced antibiotic-induced relapse of *C. difficile* infection, contained large fractions of *Lachnospiraceae* (11% compared to no detection before the first FMT and on average 1% in other RCDI samples) and *Akkermansia* (60% compared to on average 0.1% in other RCDI samples and 1.8% in healthy donor samples) (Fig. S3). This atypical composition could be responsible for the clustering of this sample with healthy donor and post-FMT patient samples in the weighted UniFrac analysis (Fig. 4B). It is therefore possible that the reductions in *Lachnospiraceae* characteristic of the other RCDI samples, rather than being a cause of disease susceptibility, represent an effect of disease duration and number of antibiotic treatment regimens exceeding those that patient #6b experienced after recurrence. Interestingly, *Akkermansia* spp. have recently received special attention in human microbiome research because of their ability to colonize the intestinal mucosa and to utilize mucus as a sole carbon and nitrogen source [57], [58]. While *A. municiphila* has been proposed as a marker of a healthy intestine, due to its production of short chain fatty acids and its negative correlation with inflammatory bowel diseases, appendicitis and obesity (reviewed here:[58]), its high abundance in the fecal sample of patient #6b might also be an indicator of high concentrations of mucus in the stool, which could be the result of acute diarrhea.

### The fecal microbiota continues to change in asymptomatic post-FMT patients

**Figure 7:**
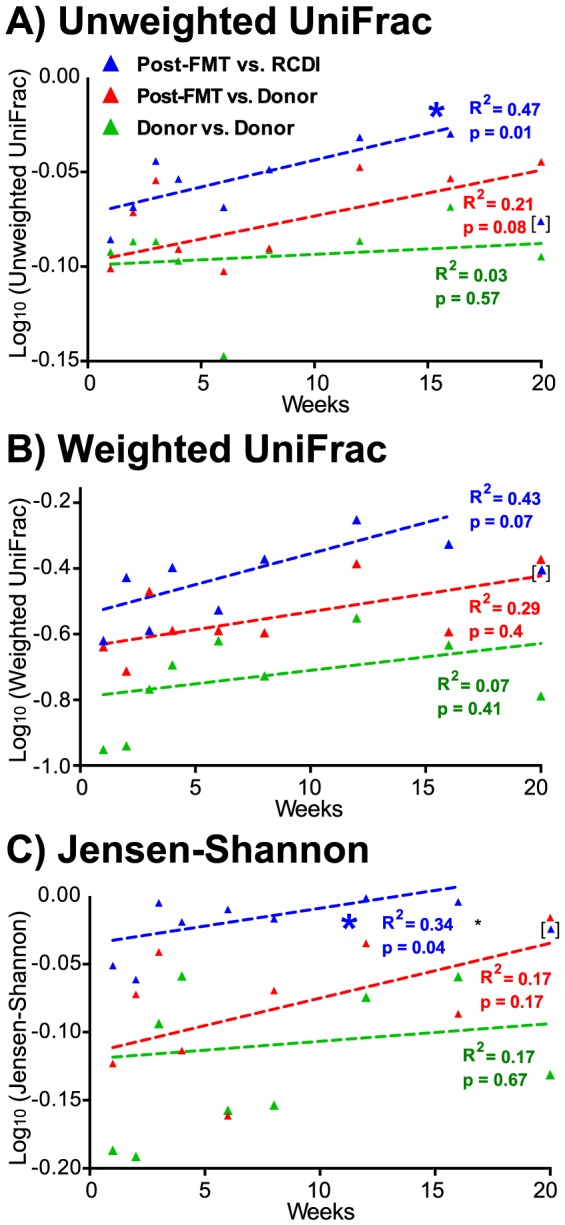
Post-FMT microbiota changes. Unweighted (A) and weighted (B) UniFrac distances and Jensen-Shannon divergence (C) metrics were calculated between post-FMT and RCDI patient sample pairs (red), post-FMT patient and donor sample pairs (green) and between donor sample pairs collected over time (blue) and plotted on logarithmic scales. R^2^ values and p-values to establish whether the slope of the curve was significantly different from zero are shown with asterisks indicating significance (p<0.05, *F*-test). The 20-week data point of patient #8 was classified as outlier and not included in the analyses, based on the Bonferroni-adjusted outlier test, and is shown with parentheses. One-year time points (patient and donor #1) were also classified as outliers and omitted from the analysis and plot. A plot showing all data points including those omitted is part of the supplement (Fig. S4).

Asymptomatic post-FMT patients appear to be at higher risk for recurrence of *C. difficile* infection compared to patients without a history of RCDI, if additional antibiotic medication to treat unrelated infections becomes necessary [27]. Whether specific microbiota features, such as the increased abundance of *Streptococcus* in post-FMT patient compared to healthy donor samples, are responsible for this susceptibility is unknown, but the susceptibility of post-FMT patients to RCDI may decrease over time and little is known about the long-term dynamics of FMT-induced microbiota changes. In order to characterize microbiota changes after FMT over time, fecal samples from post-FMT patients, all of which were asymptomatic with respect to RCDI, were compared longitudinally. Microbiota diversity in post-FMT patient samples did not change significantly over time, as measured by comparing the Shannon diversity index (Fig. S1). To study changes in microbiota composition over time, weighted and unweighted UniFrac distances and the Jensen-Shannon divergence were calculated between (i) RCDI and post-FMT patient sample pairs, (ii) donor and post-FMT patient samples pairs and, as a control for temporal variations in healthy individuals, between (iii) sample pairs collected from the same donor before and after FMT (Fig. 7). For the comparison of post-FMT and RCDI patient samples, both unweighted UniFrac and Jensen-Shannon distance metrics displayed a significant linear change over time when plotted on a logarithmic scale. However, comparison of post-FMT patient and donor samples or of donor samples collected before and after FMT did not. That this correlation is only apparent if temporal changes are plotted on a logarithmic scale shows that the most significant changes happen immediately after FMT and that the microbiota continues to evolve over time albeit at a decreasing rate.

**Figure 8:**
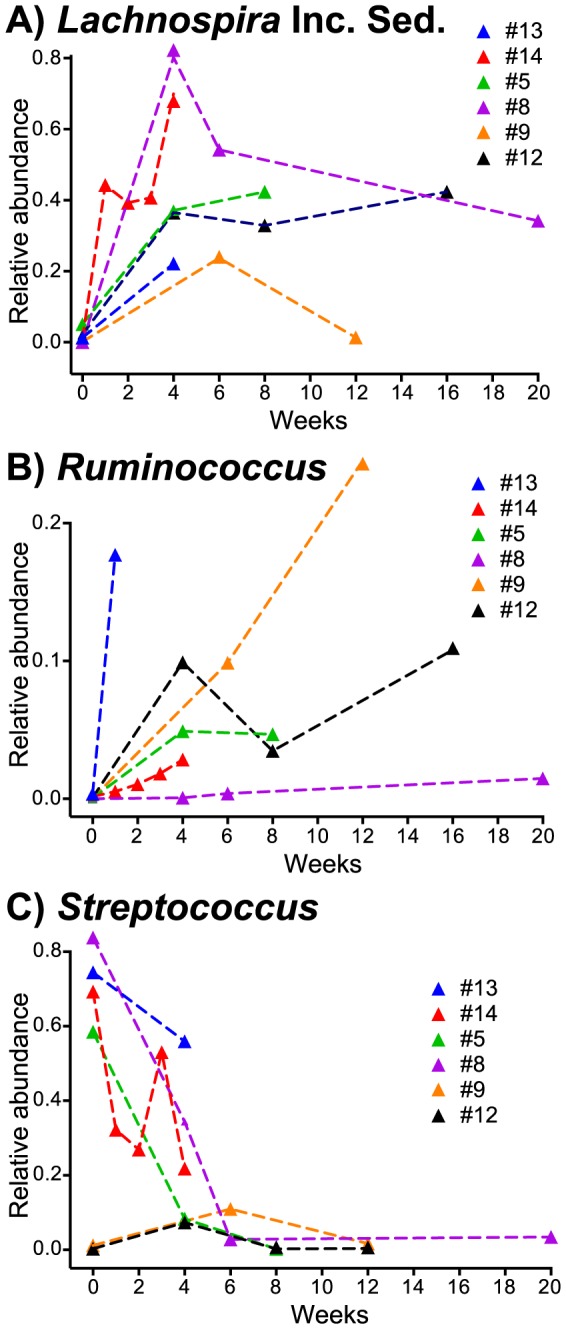
Post-FMT changes in selected microbiota members by case (genus level). (A) *Lachnospira* Incertae Sedis; (B) *Ruminococcus*; (C) *Streptococcus*. Genus-specific changes in relative abundance over time were not significant (p>0.05)when samples were grouped by time periods (1 week, 2–4 weeks, 6–8 weeks, 12–20 weeks) and groups compared with a non-parametric statistical test (Wilcoxon rank sum test).

Individual taxonomic families showed similar trends in post-FMT patients over time, if compared case-by-case, i.e. increases in *Lachnospiraceae* and *Ruminococcaceae* and decreases in *Streptococcaceae* (Fig. 8). However, in contrast to changes in relative abundance between the pre- and post-FMT patient microbiota (Fig. 6), changes in post-FMT patients over time were not significant for the three studied Firmicutes families. This suggests that, while changes in the abundance of *Lachnospiraceae* and/or *Streptococcaceae* might play important roles for RCDI or successful recovery after FMT in some patients, general post-FMT microbiota dynamics across the entire patient population are better described using metrics that take account of the microbiota as a whole, i.e., UniFrac distances and Jensen-Shannon divergence.

### ‘Keystone’ species are not identified in RCDI or FMT

The concept of keystone species has been used to describe the disproportionate importance of a single or a few organisms for the structure or function of an entire environment [59], [60], e.g. in the oral cavity where colonization with the commensal bacterium *Porphyromonas gingivalis* even at low abundance can play a major role for microbiota changes associated with periodontitis [61]. In the context of RCDI and FMT, keystone bacteria could be crucial for the identification of diagnostic markers to predict susceptibility to *C. difficile* infection and as substitutes for fecal samples of largely unknown composition to be used in transplantation. That RCDI can principally be treated by transplantation of *in vitro*-assembled microbial communities instead of fecal material was shown recently in humans [54] and mice [52], although little justification was provided for the selection of specific bacterial species or strains. While, based on our findings and previous data, members of the *Lachnospiraceae* family, for example, might present themselves as keystone candidates [50], [62], [63], at least one case was found in our cohort where RCDI was associated with relatively high counts of *Lachnospiraceae* (i.e., #6b). In another case (#9), *Lachnospiraceae* did only increase temporarily six weeks after FMT but dropped to pre-FMT levels 12 weeks after FMT. Khoruts et al. found a relatively large proportion (>5%) of *Lachnospiraceae* Inc. Sed. in an RCDI sample before FMT treatment [25]. Interestingly, the dominant representative of the genus *Lachnospiraceae* Inc. Sed. associated with successful FMT treatment, which was identified in the Canadian study by Shahinas et al. [50], is different from the one identified here (Shahinas: 97% identical to GenBank Acc.-No. JX230866, compared to this study: 99% identical to EF399262). This difference could either result from variations in the applied pyrosequencing protocols (e.g., Shahinas et al. used primers specific for hypervariable regions V5–V6 instead of primers specific for V1–V3 used here) or indicate that different species or strains of the genus *Lachnospiraceae* Inc. Sed. circulate in U.S. and Canadian human populations. In any case, it seems as if neither RCDI nor FMT are associated with the presence or absence of a single specific microbiota fraction.

Instead of bacterial keystone taxa, specific microbial microbiota genes or transcripts could be associated with health and disease and, thus, serve as "keystone functions" with potential as diagnostic markers. A redundancy and similarity of functional microbiota compositions between individuals despite significant taxonomic variation has previously been demonstrated for the healthy human microbiota [64]. These functions could be predominantly but not exclusively associated with certain members of the fecal microbiota, which would then still show statistical correlations with health and disease states. Short-chain fatty acid (SCFA) production plays an important role in the regulation of intestinal inflammatory processes [65] and intestinal barrier maintenance [66]–[68] and has been discussed in the context of RCDI, as *C. difficile* infection in the mouse model was shown to alter SCFA profiles [52]. Consequently, the reduction of *Lachnospiraceae* and *Ruminococcaceae* has been interpreted as a depletion in butyrate-producing bacteria [51]. Shotgun sequencing of total metagenomic DNA and/or metatranscriptomic RNA isolates will be needed to confirm the lack of butyrate production in the fecal RCDI microbiota or to associated other "keystone functions" with RCDI and FMT.

### Concomitant effects of antibiotics and diarrhea

Previous RCDI microbiota studies have had difficulty determining the chain of events leading to disease as well as the relationship between observed microbiota phenotypes and disease. *C. difficile* infection is typically initiated by antibiotic treatment and phenotypically characterized by severe diarrhea. Both events by themselves have a massive impact on the fecal microbiota independent of the disease caused by the *C. difficile* infection [69], [70]. It is therefore difficult to distinguish between microbiota changes that play a causative role in RCDI and those that simply co-occur. The data presented here also include an RCDI patient with successful FMT and subsequent relapse of CDI after antibiotic treatment, whose fecal microbiota showed characteristics described for healthy individuals as opposed to RCDI patients (e.g. relatively high *Lachnospiraceae* abundance). This single patient may therefore suggest that multiple rounds of antibiotic treatment and/or long-term duration of the disease are needed to induce some of the microbiota changes previously reported to be associated with CDI. In order to determine the exact time line of events, prospective studies are needed starting before antibiotic treatment and following patients during the onset and course of CDI.

## Conclusion

In accordance with previous reports, we found a reduction in microbiota diversity and richness in fecal samples from RCDI patients compared to healthy donors, which was restored after FMT. Similarly, our results confirm previous findings that FMT changes the RCDI fecal microbiota to become more similar to that of healthy donors. We extend current knowledge by demonstrating that there are different varieties of dysbiosis in RCDI patient samples, that FMT predominantly affects Firmicutes and Proteobacteria, and that the fecal microbiota continues to change in post-FMT patients. We did not identify a ‘keystone’ species in RCDI or FMT, but our findings suggest that butyrate producing bacteria may be important. We believe that additional longitudinal studies, ideally beginning before initial infection and including metagenomic and metatranscriptomic analyses, will lead to improved outcomes in *C. difficile* infection.

## Supporting Information

Figure S1 Fecal microbiota diversity in patient and donor samples depending on collection time points. The Shannon index of all samples is plotted over time, split into donor (A, blue) and patient (B, red) samples.
(PDF)

Figure S2 Venn diagram showing shared OTUs between RCDI and post-FMT patient and donor samples. Only OTUs represented by at least 5 reads across all 56 samples are shown.
(PDF)

Figure S3 Microbiota changes between RCDI samples collected from the same patient before the first FMT (#6a) and, after antibiotic-induced relapse, before the second FMT (#6b). Relative abundances of all taxonomic genera (>1%) are shown.
(PDF)

Figure S4 Post-FMT microbiota changes. Unweighted (A) and weighted (B) UniFrac distances and Jensen-Shannon divergence (C) metrics were calculated between post-FMT and RCDI patient sample pairs (red), post-FMT patient and donor sample pairs (green) and between donor sample pairs collected over time (blue). This figures shows that both patient and donor samples from case #1 collected one year aft FMT show an unusual small divergence (Unweighted/weighted UniFrac distances and Jensen-Shannon divergence) from the donor sample collected before FMT.
(PDF)

Table S1 Numbers of reads and identified operational taxonomic units (OTUs) by sample.
(XLSX)
