# Correction: Microbiota Dynamics in Patients Treated with Fecal Microbiota Transplantation for Recurrent *Clostridium difficile* Infection

**DOI:** 10.1371/journal.pone.0104471

**Published:** 2014-07-29

**Authors:** The PLOS ONE Staff

In the second paragraph of the “Amplifications and sequencing” section of the Materials and Methods, the SRA number is incorrect. The corrected sentence should read: “Raw sequences were deposited in the Short Read Archive Database (http://www.ncbi.nlm.nih.gov/sra; project number SRP030777).”
