# Supplementary material for: Microbiota Dynamics in Patients Treated with Fecal Microbiota Transplantation for Recurrent Clostridium difficile Infection
Source: PLoS One. 2013 Nov 26;8(11):e81330. doi: 10.1371/journal.pone.0081330 (PMC3841263; doi:10.1371/journal.pone.0081330)
Supplement: Figure S1 — Fecal microbiota diversity in patient and donor samples depending on collection time points. The Shannon index of all samples is plotted over time, split into donor (A, blue) and patient (B, red) samples. (PDF) [file pone.0081330.s001.pdf]

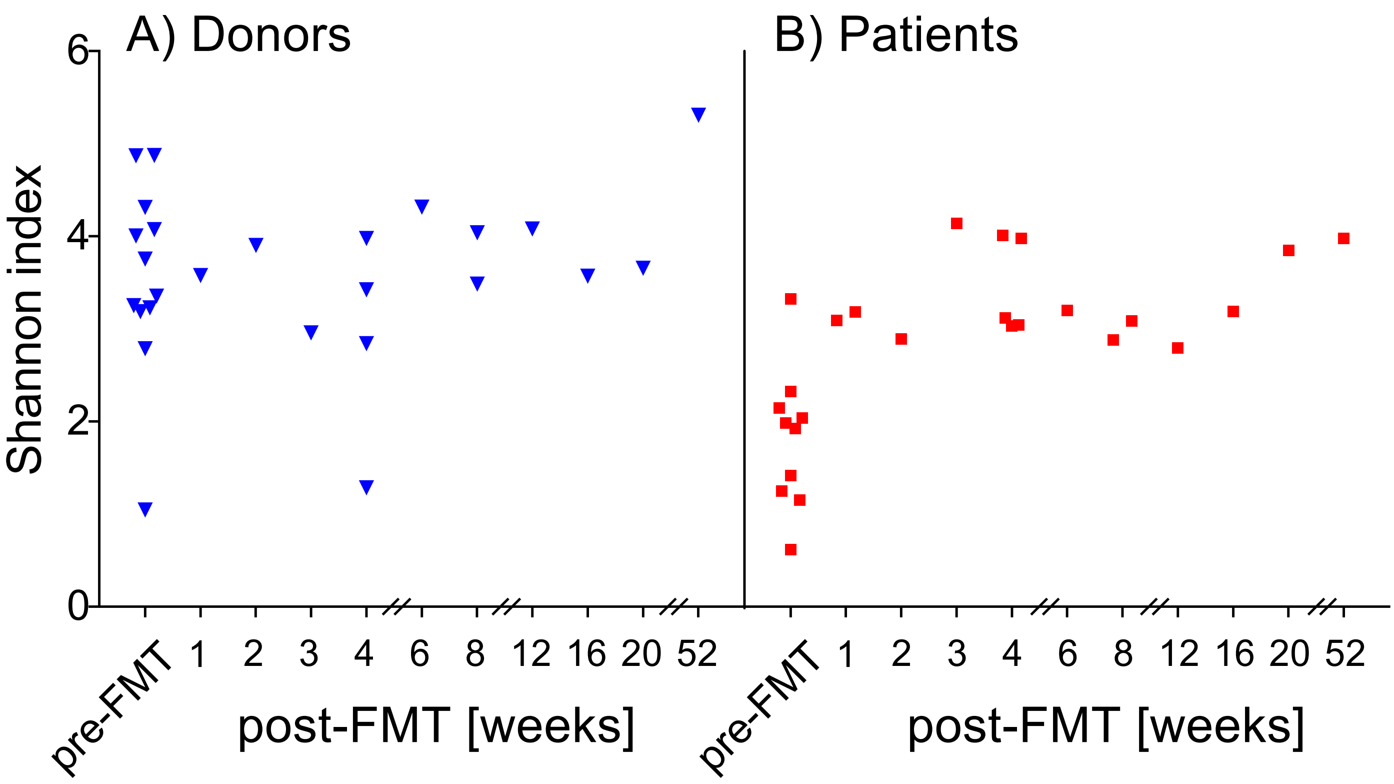

Figure S1. Fecal microbiota diversity in patient and donor samples depending on collection time points. The Shannon index of all samples is plotted over time, split into donor (A, blue) and patient (B, red) samples.
