# Supplementary material for: Microbiota Dynamics in Patients Treated with Fecal Microbiota Transplantation for Recurrent Clostridium difficile Infection
Source: PLoS One. 2013 Nov 26;8(11):e81330. doi: 10.1371/journal.pone.0081330 (PMC3841263; doi:10.1371/journal.pone.0081330)
Supplement: Figure S2 — Venn diagram showing shared OTUs between RCDI and post-FMT patient and donor samples. Only OTUs represented by at least 5 reads across all 56 samples are shown. (PDF) [file pone.0081330.s002.pdf]

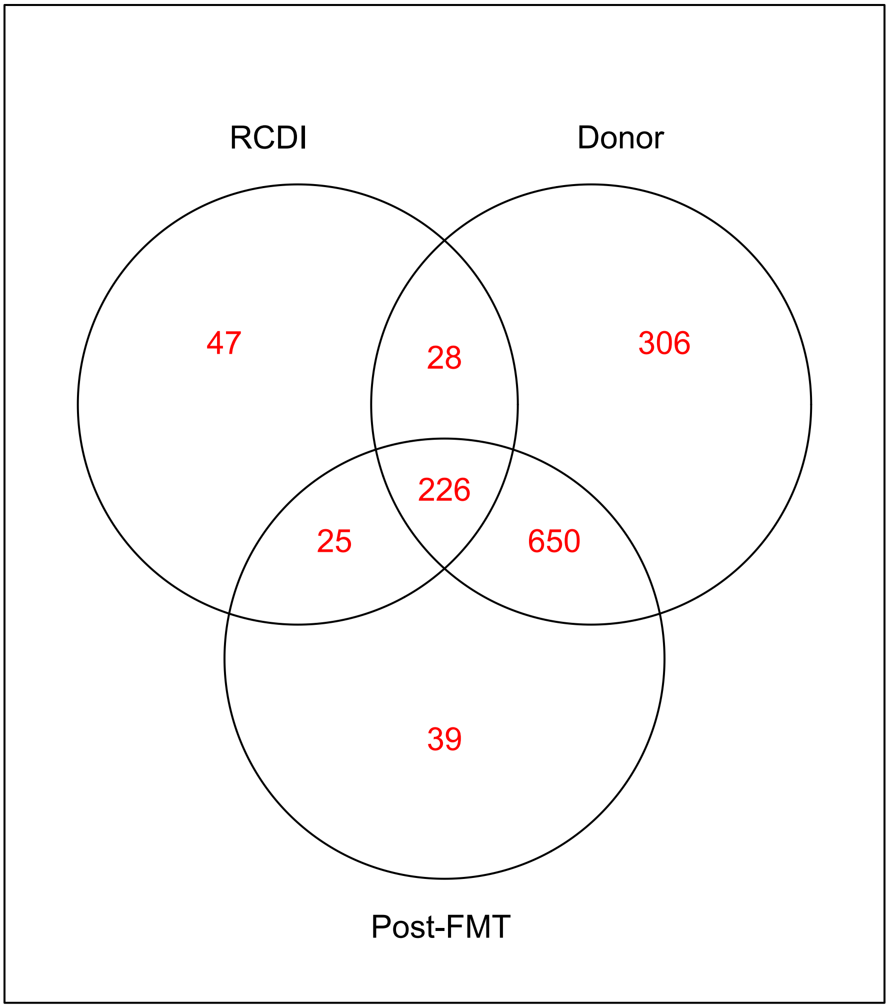


**Figure S2. Venn diagram showing shared OTUs between RCDI and post-FMT patient and donor samples.** Only OTUs represented by at least 5 reads across all 56 samples are shown.
