# Supplementary material for: Microbiota Dynamics in Patients Treated with Fecal Microbiota Transplantation for Recurrent Clostridium difficile Infection
Source: PLoS One. 2013 Nov 26;8(11):e81330. doi: 10.1371/journal.pone.0081330 (PMC3841263; doi:10.1371/journal.pone.0081330)
Supplement: Figure S3 — Microbiota changes between RCDI samples collected from the same patient before the first FMT (#6a) and, after antibiotic-induced relapse, before the second FMT (#6b). Relative abundances of all taxonomic genera (>1%) are shown. (PDF) [file pone.0081330.s003.pdf]

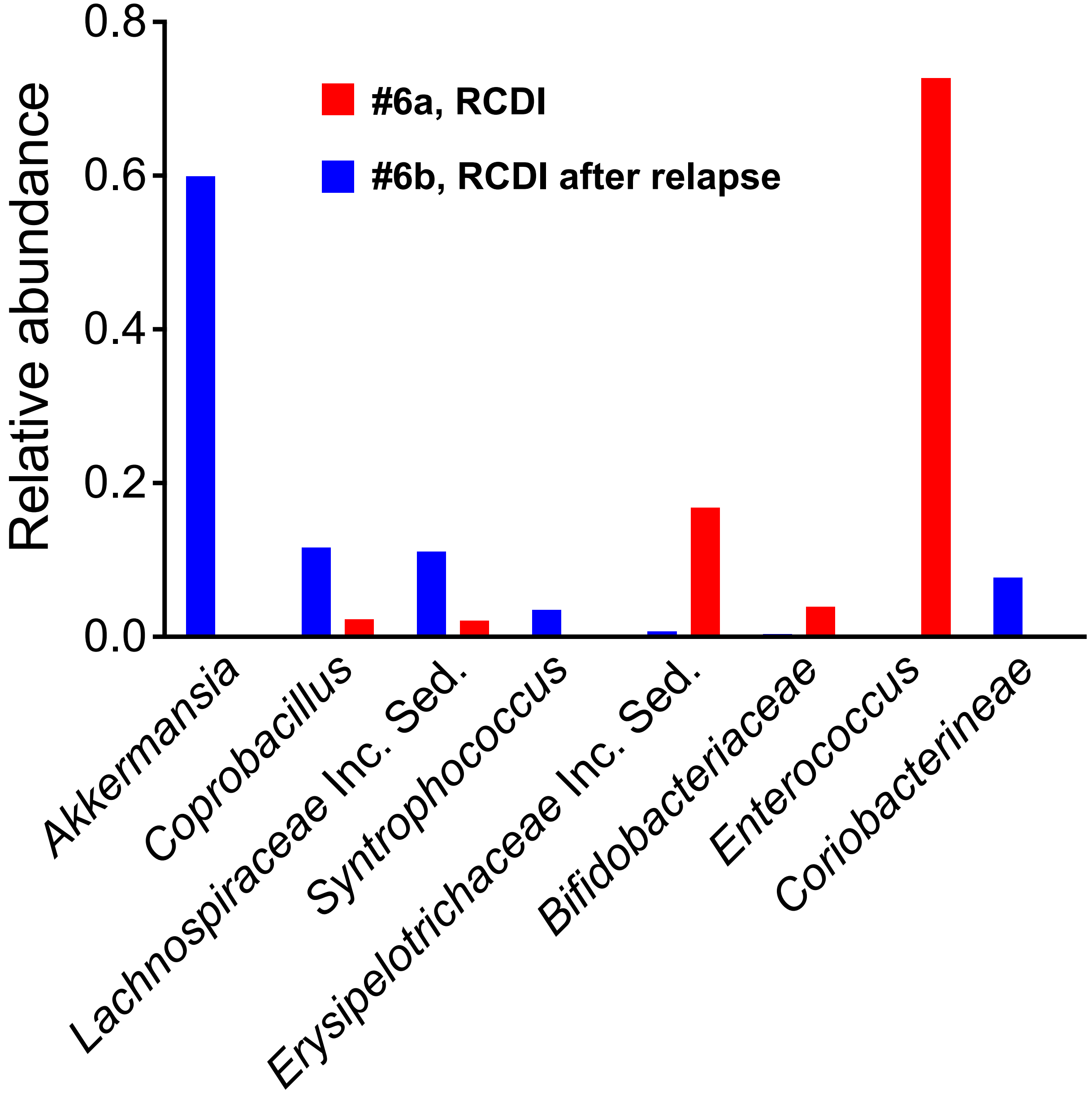

Figure S3. Microbiota changes between RCDI samples collected from the same patient before the first FMT (#6a) and, after antibiotic-induced relapse, before the second FMT (#6b). Relative abundances of all taxonomic genera (>1%) are shown.
