# Supplementary material for: Microbiota Dynamics in Patients Treated with Fecal Microbiota Transplantation for Recurrent Clostridium difficile Infection
Source: PLoS One. 2013 Nov 26;8(11):e81330. doi: 10.1371/journal.pone.0081330 (PMC3841263; doi:10.1371/journal.pone.0081330)
Supplement: Figure S4 — Post-FMT microbiota changes. Unweighted (A) and weighted (B) UniFrac distances and Jensen-Shannon divergence (C) metrics were calculated between post-FMT and RCDI patient sample pairs (red), post-FMT patient and donor sample pairs (green) and between donor sample pairs collected over time (blue). This figures shows that both patient and donor samples from case #1 collected one year aft FMT show an unusual small divergence (Unweighted/weighted UniFrac distances and Jensen-Shannon divergence) from the donor sample collected before FMT. (PDF) [file pone.0081330.s004.pdf]

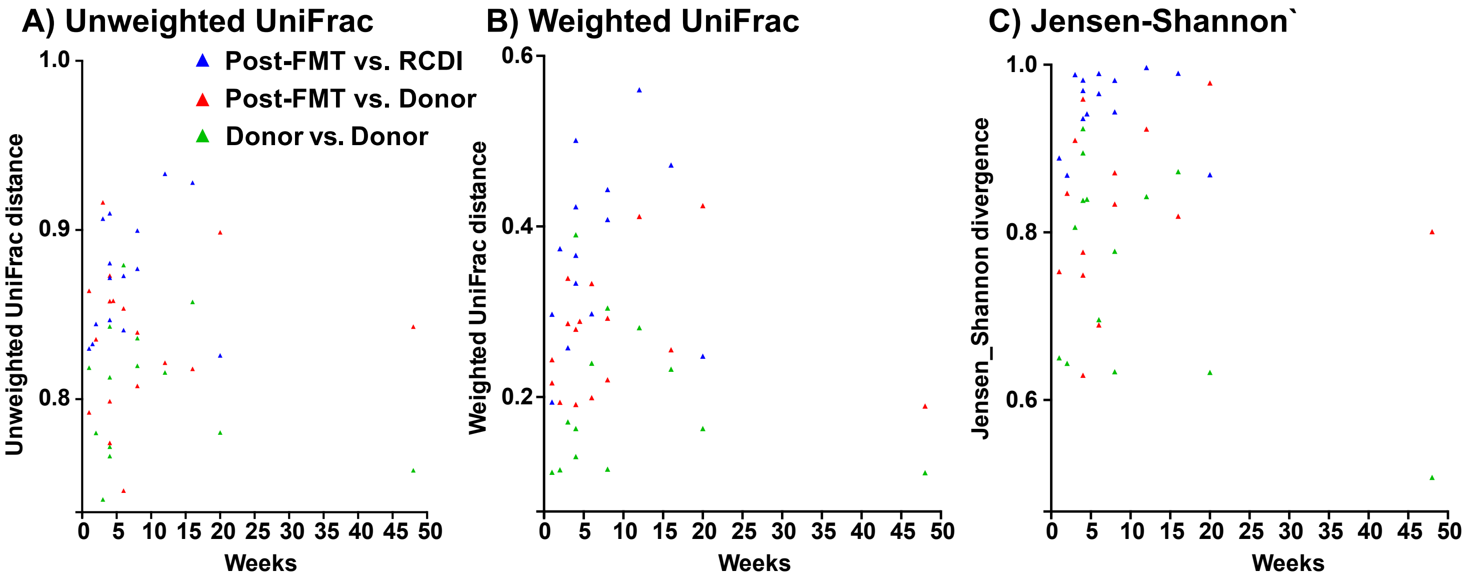

Figure S4. Post-FMT microbiota changes. Unweighted (A) and weighted (B) UniFrac distances and Jensen-Shannon divergence (C) metrics were calculated between post-FMT and RCDI patient sample pairs (red), post-FMT patient and donor sample pairs (green) and between donor sample pairs collected over time (blue). This figures shows that both patient and donor samples from case #1 collected one year aft FMT show an unusual small divergence (Unweighted/weighted UniFrac distances and Jensen-Shannon divergence) from the donor sample collected before FMT.
